# Forming Quality Research on the Variable-Diameter Section of the Hollow Axle in Three-Roll Skew Rolling

**DOI:** 10.3390/ma15165614

**Published:** 2022-08-16

**Authors:** Yingxiang Xia, Xuedao Shu, Jianan Shi, Ying Wang, Zbigniew Pater, Jitai Wang

**Affiliations:** 1 Faculty of Mechanical Engineering and Mechanics, Ningbo University, Ningbo 315211, China; 2 Zhejiang Key Laboratory of Part Rolling Forming Technology, Ningbo University, Ningbo 315211, China; 3 Faculty of Mechanical Engineering, Lublin University of Technology, 20-618 Lublin, Poland

**Keywords:** three-roll skew rolling, variable-diameter section, hollow axle, forming quality

## Abstract

The hollow axle is the key component of the high-speed train, and the realization of high-quality forming is the key to ensure the safety of train operation. In this paper, the specimen of the variable-diameter section of the hollow axle is taken as the research object, and the generation mechanism of surface spiral mark defects and the formula of spiral mark depth of variable-diameter section in the TRSR (three-roll skew rolling) process with variable roll spacing are explored. The external roundness error and the function C to measure the wall thickness uniformity of the cross-section were taken as the evaluation indexes and the single-factor simulation experiment was established and simulated in the software Simufact.forming16.0 to obtain the influence law of each process parameter on the external roundness error and wall thickness uniformity of the rolled piece. Orthogonal tests were designed and the order and optimal combination of process parameters on the forming quality were obtained by range analysis and ANOVA analysis. The research results provide theoretical guidance for improving the forming quality of the variable-diameter section of the hollow axle in three-roll skew rolling, and promote the transformation of the TRSR process to high performance and accurate forming.

## 1. Introduction

The hollow axle is a new type of axle structure, which is mainly used for high-speed trains with high heavy loads due to its low spring mass, lightweight components and vibration shock reduction. At present, the hollow axle billet includes precision forging and cross-wedge rolling technology. The special roll, multi-wedge synchronous rolling [1] and headless rolling technology [2] greatly ensure the production efficiency and material utilization rate. However, heavy load pressure and custom roll lead to a high tooling cost and storage expense for production equipment [3,4].

In recent years, the three-roll skew rolling of shaft parts has become a new research direction. It is a flexible forming technology which can realize load reduction and die universality, and is widely used in the forming of seamless steel pipe [5], thread pipe [6], stepped shaft and other pipe shaft parts. The United States put into operation the world’s first Assel-type three-roll skew rolling mill in 1935, and then the mill developed rapidly because of its stable piercing ability for thick-walled parts [7,8,9,10]. Z. Pater et al. of the Lublin University of Technology innovatively manufactured a CNC three-roll skew rolling mill with variable roll spacing [11,12], and obtained the conclusion that three-roll skew rolling can be used to produce variable cross-section shaft parts such as long shaft parts, hollow stepped shafts and crankshaft preforms [13,14,15]. Meanwhile, they conceived a concept of manufacturing a hollow axle using three skewed rolls moving at the same rotating speed, an axially moving chuck and a moving mandrel [16]. The feasibility [17] and the microstructure [18] of the TRSR hollow axle were also systematically studied by Ningbo University. It was concluded that three-roll skew rolling is an advanced process for the hollow axle of high-speed trains. Shu et al. [19] revealed the microstructure evolution of forming parts at each deformation stage via analyzing the effect of process parameters on the average grain size. Hu [20] effectively simulated the microstructure evolution process of a Ti-6Al-4 V bar by three-high skew rolling technology in Deform-3D, and reasonably explained that the actual grain size is a little coarser than the CA model. At the same time, Xia innovatively applied the three-roll skew rolling process to the reduction in diameter and end thickening of aviation thin-walled tubes of aluminum alloy and expounded its thickening mechanism [21]. An innovative flexible skew rolling (FSR) with increased roll freedom was also proposed and verified by Lin. FSR can be used to manufacture large shafts with multiple specifications and small batch by programming four stages of motion, namely, radial rolling, roll tilt, skew rolling and roll straightening [22,23].

There is also a series of research on the core defects and surface quality defects of hollow axle produced by three-roll skew rolling. The main manifestations are internal fracture and surface spiral marks, which will reduce the mechanical properties of the hollow axle and increase the amount of subsequent machining and waste materials. Based on the predictability evaluation of the conventional internal fracture ductile fracture criterion of three-roll skew rolling, Yamane [24] proposed a new ductile fracture criterion for skew rolling that is specific to skew rolling and regardless of the number of rolls. Pater [25] calculated the spiral marks’ height in the reducing section of a three-roll skew rolling truck axle, and concluded that the ratio of axial chuck speed and radial feed speed is the main factor affecting the depth of spiral marks. He also predicted ductile fracture during skew rolling, and found that two-roller skew-rolled bars are more likely to fracture in their axial zone, suggesting the use of classical fracture criterion to predict cracking during skew rolling [26]. Hwang [27] discussed the influence of forming angle, deflection angle, roll shape and other factors on the depth of spiral marks on the rolled piece surface and rolling force by finite element simulation method, carried out rolling experiments on the rod made of rubber mud with different roll shapes on the independently developed three-high planetary mill and analyzed the influence law of roll shape on the depth of spiral marks. Zhang [28] discussed the formation mechanism of spiral cracks on the surface of rolled piece and optimized the spiral marks by controlling the roll speed.

The above-mentioned research systematically analyzed the deformation mechanism and innovative application in the forming process of three-roll skew rolling, but there is little research on the forming law of the variable-diameter section with variable roll spacing. The billet size, die size and process parameters will affect the state of the rolled piece and thus affect the final processing size. In this study, the forming law of the variable-diameter section of the hollow axle in three-roll skew rolling is studied. Through the kinematic analysis, the generation mechanism of the surface spiral mark defect and the spiral mark depth are explored. Combined with the finite element simulation, the existing problems are analyzed and the optimization measures are given, which provides a theoretical guidance for improving the forming quality of the variable-diameter section of the three-roll skew rolling, and has important engineering significance for further promoting the application of the three-roll skew rolling forming of hollow long axle parts.

## 2. Finite Element Model and Experiment Verification

**Figure 1:**
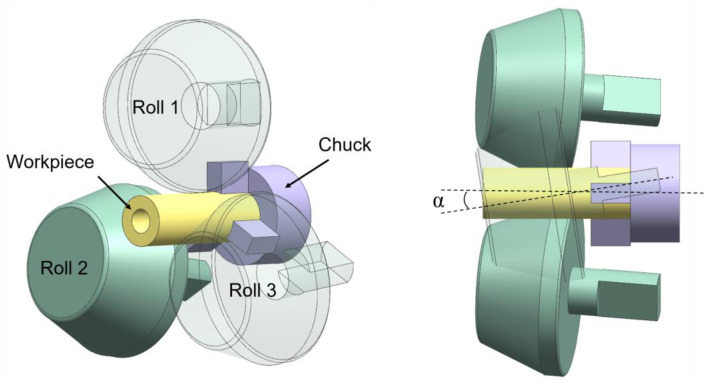
Finite element model.

The assembly consisting of three centrally symmetrical rolls, chucks and blank established in NX.10.0 was exported to the stl. format and imported into the Simufact.Forming16.0 hot rolling process. The speed drive table and motion freedom of each moving part were set according to the shape of the rolled piece and the process parameters. The forming characteristics of the TRSR with variable roll spacing under local loading needed concentrated stress. The roll was designed as a disc roll with an inclined roll surface and short cylindrical surface with a finishing section length of 20 mm. In order to reduce the wear of the finishing area and improve the finishing effect, the tail fillet was 2.5 mm. This disc roll ensured the roll effect under the premise of small size, and realized the lightweight processing of the rolling mill by local loading. The established finite element model is shown in Figure 1.

Since rolling is a complex forming process with multi-variable nonlinearity and elastic–plastic composite of materials, the simulation process is simplified based on the professional plastic forming simulation software Simufact.Forming16.0.

1. The roll and chuck were set as rigid bodies with initial temperatures of 150 °C, and the billets were set as rigid-plastic bodies and divided by tetrahedral meshes with a size of 3 mm;
2. The shear friction model suitable for large deformation (friction coefficient 0.8) was adopted for the contact between the rolled piece and the roll. The bonding between the rolled piece and chuck was set to ensure that the contact surface was not separated during the rolling process (friction coefficient 1), and the clamping position of the chuck was optimized to be circular to avoid the local large stress caused by linear contact;
3. The convective heat transfer coefficient was 200 W mm^−2^ K^−1^, and the heat conductivity was 2 × 10^4^ W mm^−2^ K^−1^;
4. The cylindrical billet of 30CrMoA alloy steel with an outer diameter of 52 mm and inner diameter of 25 mm was selected. Its phenomenological constitutive equation [29] was imported into the software material library to generate the high temperature rheological curve of 30CrMoA at 950–1150 °C, as shown in Equation (1)

(1) $$\dot{\varepsilon }=3.67\times 10^{9}{\left[\sinh\left(0.011\sigma_{p}\right)\right]}^{4.403}\exp\left[-261850/(RT)\right]$$

where *R* is the ideal gas constant (8.3145 J·mol^−1^ K^−1^), *T* is the deformation temperature (K) and $\dot{\varepsilon }$ is the material strain rate (s^−1^).

**Figure 2:**
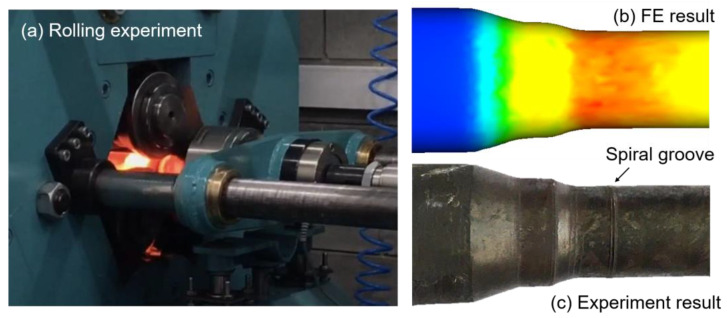
Comparison of FE and experiment results.

In order to verify the reliability of the finite element model, it was necessary to carry out a rolling experiment for the variable-diameter section of the hollow axle. The rolling equipment was Lublin University of Technology’s variable roll pitch three-roll skew rolling mill. The equipment was a trial rolling machine for the production of reduced proportional shaft, and it was suitable for small piece rolling. When heating the billet to 1150 °C, it should be noted that it still takes time for the rolled piece to be clamped from the box heating furnace to the rolling mill. The heating temperature of the heating furnace needs 50 °C higher. Figure 2 is the FE simulation and rolling experiment results under same process parameters, which show that the shape of the rolled piece was basically similar.

**Figure 3:**
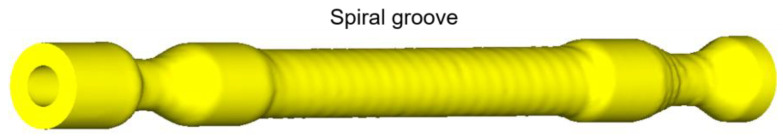
Spiral groove.

Spiral marks are the most common surface quality problems in the traditional three-roll skew rolling process, as shown in Figure 3. The essence of spiral marks is the three-end spiral track of the roll contact area acting on the rolled piece. The rolling pitch of the roll greatly affects the forming accuracy of the variable-diameter section. The ideal rolling process is that the surface metal of the rolled piece moves radially internal or axially through the extrusion of the roll, so as to form the contour of the trajectory streamline of the mold edge motion. However, the selection of process parameters will directly affect the streamline shape of the roll trajectory. Usually, the length of the finishing section can be added by enhancing the roll surface length to increase the contact area, which can effectively reduce the depth of the spiral mark.

## 3. Theoretical Analyses of Variable-Diameter Section

**Figure 4:**
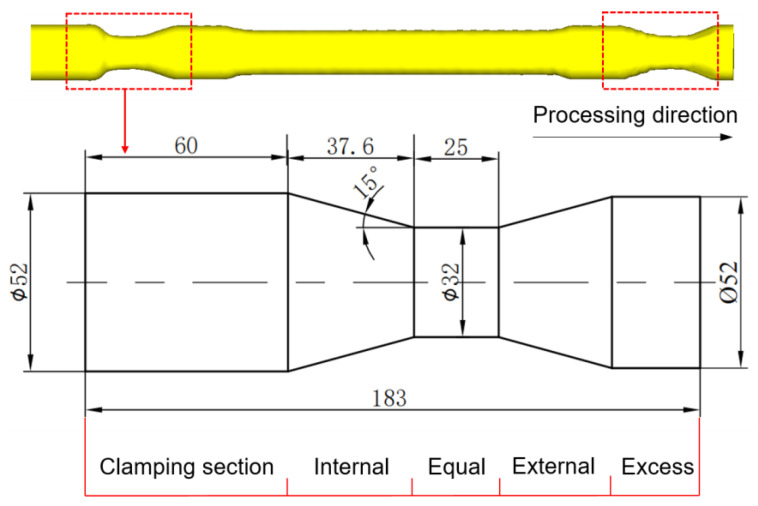
Simplified model of variable-diameter section.

The three-roll skew rolling hollow axle can be simplified into three typical rolling processes: equal diameter and internal and external variable-diameter rolling sections. According to the section change, the axle is divided into the clamping section, internal variable-diameter, external variable-diameter and excess section along the rolling direction, as shown in Figure 4. The research on the variable-diameter section based on the segmentation can simplify the model to the maximum extent and reduce the simulation time cost. The large-scale variable-diameter section near the two ends of the axle was selected as the test specimen for research.

### 3.1. Accuracy of Internal Variable-Diameter Section

**Figure 5:**
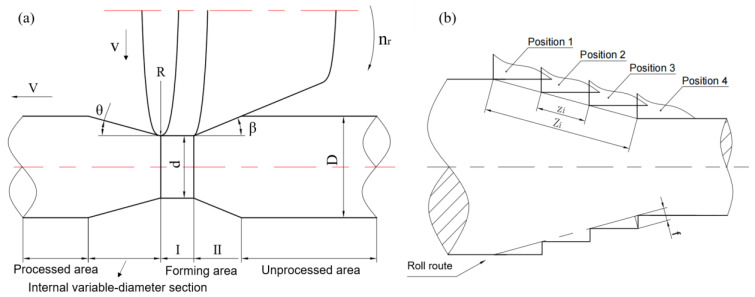
The internal variable-diameter section (**a**) forming stage and (**b**) lead analysis of processing path.

The internal variable-diameter section is the stage of processing to a smaller diameter section. In this stage, the round chamfer at the tail of the roll is used as the processing wedge to squeeze the metal to the front, so as to form the contour of the stepped shaft. The schematic diagram of the rolled piece at *t* in the rolling process of the internal variable-diameter section is shown in Figure 5a. The billet diameter is *D*, which is drawn by the chuck at a fixed axial speed *V*. The roll rotates at a fixed speed *n_r_* and feeds at a constant rate *v* in the radial direction of the rolled piece. The angle between the roll surface and the axial direction is *β*. Through the coordination of the roll *v* and the chuck *V*, the internal variable-diameter section with an angle of *θ* can be formed, and the minimum section diameter of the variable-diameter is *d*. This stage of the rolled piece is divided into five parts for analysis, which, from left to right, are processed area, internal variable-diameter section, forming areas I (equal diameter) and II (variable-diameter) and unprocessed area. The processing path of the mold moving along the rolling route is shown in Figure 5b (mold position 1–4), and the saw tooth defect will occur when the elastic deformation is ignored.

The surface contact between the rolled piece and the roll is dominant in the process of internal variable-diameter forming, and the radius of different contact points on the roll is different, which will lead to the difference of point linear velocity. Since the finishing section of the roll is always close to the rolled piece in the process of forming the internal variable-diameter section, the local slip is ignored here. The rotational speed of the rolled piece is calculated based on the linear velocity of the finishing section of the roll, and then the velocity transmission can obtain:

(2) $$n_{w}=\frac{2Rn_{r}}{d},$$

where, *n_r_* is the roller speed, *n_w_* is the workpiece speed, *d* is the nominal diameter of the rolled piece corresponding to the finishing section of the roller and *R* is the roller radius. The workpiece speed will gradually increase with the continuous decrease in the *d* of the internal variable-diameter section. When calculating the single lead and pitch, the diameter change is ignored, and the time of the rolled piece rotating for a week can be expressed as *t_i_*:

(3) $$t_{i}=\frac{60}{n_{w}}=\frac{30d}{Rn_{r}}$$

The rolling guidance range *Z_i_* of the internal variable-diameter section is:

(4) $$Z_{i}=\frac{30dV}{Rn_{r}\cos\theta }$$

In the formula, *V* is the axial traction speed of the chuck, *θ* is the inclined angle of the internal variable-diameter section relative to the workpiece axis, then the rolling pitch is:

(5) $$z_{i}=\frac{Z_{i}}{3}=\frac{10dV}{Rn_{r}\cos\theta }$$

From Equation (4), it can be concluded that the rolling pitch of the internal variable-diameter section is proportional to the axial speed and workpiece diameter, and inversely proportional to the roll diameter, roll speed and the inclination angle. The above rolling pitch will produce serrated contour as shown in Figure 2, the maximum size deviation of serrated *f_i_*:

(6) $$f_{i}=\frac{z_{i}\tan\theta }{\tan\theta +1}=\frac{10dV\sin\theta }{Rn_{r}\cos^{2}\theta (tan\theta +1)}$$

It can be seen that to improve the forming accuracy of the rolled piece can be directly achieved by adjusting the process parameters to reduce the surface size deviation. The above calculation is based on the case that there is no chamfer at the end of the finishing section of the roller. If the chamfer is adopted, the maximum size deviation at the saw tooth can be further reduced without changing the rolling guidance range *Z_i_* and the rolling pitch *z_i_*.

### 3.2. Accuracy of External Variable-Diameter Section

**Figure 6:**
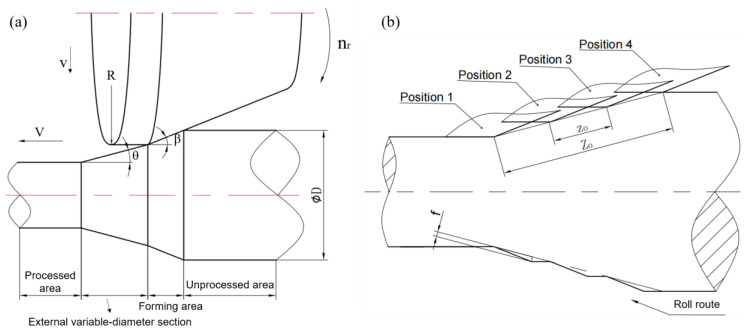
The external variable-diameter section (**a**) forming stage and (**b**) lead analysis of processing path.

The generation mechanism of spiral marks in the external variable-diameter section is different. Figure 6b shows the shape section of the rolled piece formed according to the movement route. It can be seen that surface appearance will appear when the elastic deformation of the material is ignored. Different from the internal, the forming of the external variable-diameter section is formed by the reduction section of the inclined roll surface and the fine section of the cylindrical surface, and the two surfaces have a fixed sharp angle *β*, which makes the saw tooth much smoother than that of the internal variable-diameter section. The difference in the forming mechanism also makes the forming quality significantly better than that of the internal variable-diameter section under the same process parameters.

The local slip is also ignored because the finishing section of the roll is always close to the rolled piece, and the workpiece rotational speed is calculated based on the linear velocity of the finishing section (as shown in Equation (1)). The rolling guidance range *Z_o_* of the external variable-diameter section of the three-roll skew rolling is:

(7) $$Z_{o}=\frac{30dV}{Rn_{r}\cos\theta }$$

In the formula, *V* is the axial traction speed of the chuck, *θ* is the inclined angle of the outer diameter section relative to the axis of the rolled piece, then the rolling pitch *z_o_* is:

(8) $$z_{o}=\frac{Zo}{3}=\frac{10dV}{Rn_{r}\cos\theta }$$

The existence of the above rolling pitch will produce a wavy profile as shown in Figure 2b. According to the geometric relationship, the maximum size deviation *f_o_* of the internal diameter-changing section in the forming can be calculated:

(9) $$f_{o}=\frac{z_{o}\cdot \tan\theta \cdot \tan(\beta -\theta )}{\tan\theta +\tan(\beta -\theta )}=\frac{10dV\cdot \tan\theta \cdot \tan(\beta -\theta )}{Rn_{r}\left[\tan\theta +\tan(\beta -\theta )\right]}$$

It can be seen from Equation (8) that to improve the forming accuracy and reduce the surface size deviation *f* can be directly achieved by reducing the axial speed of the chuck and increasing the roller speed.

## 4. Effect of Process Parameters on Forming Quality of Variable-Diameter Section

In order to explore the influence of various process parameters on the surface forming accuracy and roundness of the variable-diameter section, the roll speed, the axial speed of chuck and the radial reduction speed of roll were selected as variables to carry out the single-factor experiment with three factors and five levels. The workpiece initial temperature was 1100 °C, and the feed angle was 7°. Five levels of process parameters were selected, as shown in Table 1.

### 4.1. Evaluation Index of Forming Quality

**Figure 7:**
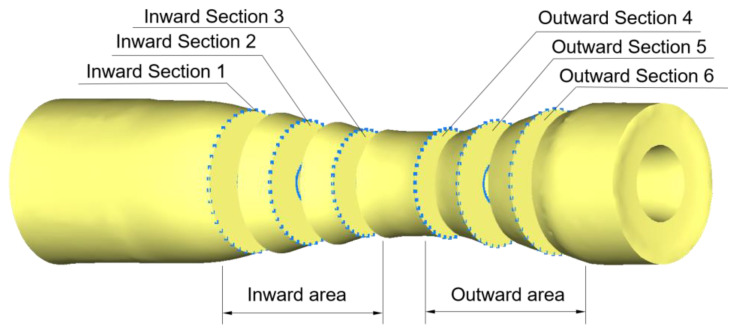
Sections and post-processing picking points.

TRSR has difficulty ensuring the core hole shape due to its local loading characteristics. The large roundness error and uneven wall thickness distribution will cause uneven circumferential strength and center of gravity deviation of the axle, and it is prone to damage under periodic stress loading during high-speed rotation. The outer roundness is the index that the outer contour surface of the rolled piece is close to the normal roundness. The cross-section of the rolled piece is taken at every interval distance in the axial direction, and six sections are taken in the internal and external variable-diameter section. The surface line is established on the outer contour of the section, and 40 points are taken on average according to the position, as shown in Figure 7.

The minimum area method is used to determine the roundness error by using the coordinate position of the points on each section: the roundness error is taken as the radius difference of the two concentric circles that contains the minimum radius difference of the measured circle contour. The greater the roundness error, the greater the distribution radius range of each point on the section, that is, the more uneven the forming size of the rolled piece on the section. At the same time, the large processing amount of subsequent turning will lead to greater vibration of the turning tool, so the roundness error reflects the surface forming quality. The formula of roundness error is as follows:

(10) $$f_{\phi }=R_{\max}-R_{\min}$$

Assuming that the diameter corresponding to the inner circle point of the cross-section is *d*_1_, *d*_2_, *d*_3_ … *d_n_*, and the diameter corresponding to the outer circle point is *D*_1_, *D*_2_, *D*_3_ … *D_n_*, the function *C* of the workpiece thickness uniformity of the cross-section is introduced, which reflects the deviation degree of the thickness of each point relative to the average value, so the more uniform the thickness of the cross-section is, the smaller the *C* value is. The more uneven the section wall thickness is, the larger the *C* value is.

(11) $$C=\frac{\sum_{i=1}^{n}(\sum_{n=1}^{n}\left|D_{i}-d_{n}-\bar{D}+\bar{d}\right|))}{n^{2}}$$

**Figure 8:**
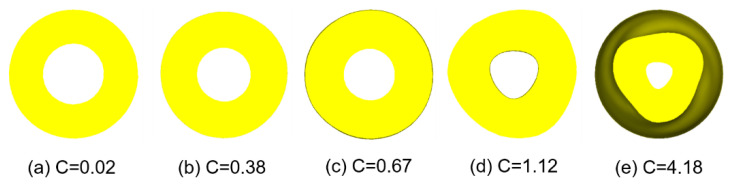
*C* value level corresponding to different cross-sections.

In order to more intuitively show the distribution of *C* function and wall thickness uniformity, several representative sections were intercepted and the corresponding *C* values were calculated. Figure 8a for unrolled section, Figure 8b–e, which are more uniform section, slightly uneven section, obvious uneven section and serious defect section after rolling, and the corresponding *C* value is shown in Figure 8. When *C* is less than 0.4, the thickness distribution of the rolled piece section is basically uniform, and there is no obvious defect. When the *C* value is greater than 1, there is a visible phenomenon of uneven wall thickness, which shows that there is a serious defect of uneven wall thickness distribution. When severe forming defects occur in the rolled piece, the *C* value reaches 4.18, indicating that the uniformity of wall thickness distribution is extremely poor at this time, and the combination of process parameters needs to be adjusted. It shows that the reasonable *C* value range to judge the thickness uniformity of cross-section is below 0.7, the lower the better.

### 4.2. Effect of Process Parameters

#### 4.2.1. Roll Rotational Speed

**Figure 9:**
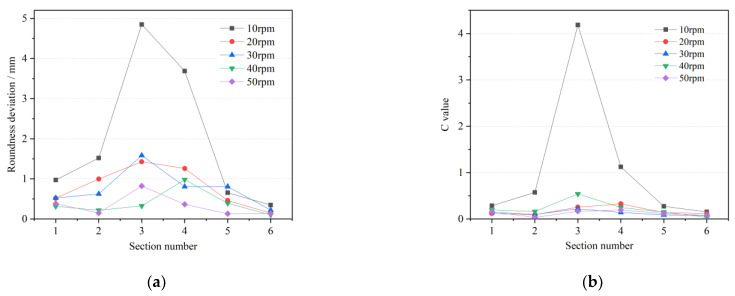
Forming quality under different roll rotational speeds (**a**) roundness deviation; (**b**) wall thickness uniformity.

Figure 9 shows the forming quality under different roll speeds. When the roll speed is 10 rpm, the roundness deviation and wall thickness uniformity of the cross-section are at large levels, and reach the maximum values in the third section with the largest feed rate. At this time, the cross-section shape is very irregular, and the wall thickness distribution is disordered due to the incomplete forming. This speed cannot be coordinated with other process parameters. With the increase in roll speed, the roundness deviation and *C* value of the cross-section decrease obviously. The rolled piece at 30 rpm is similar to that at 20 rpm, and the forming accuracy is slightly improved. When the rotational speed is further increased to 40 rpm, the roundness deviation of Section 1, Section 2 and Section 3 is less than 0.5 mm, and the combination of process parameters at this time can achieve high roundness accuracy of internal diameter variation. When the roll speed is 50 rpm, the average outer roundness deviation and *C* value of the rolled piece are the lowest, the shape is close to the positive circle and the surface forming quality is favorable. It shows that increasing the roll speed can effectively improve the outer roundness level of the variable-diameter section and improve the surface forming quality of the rolled piece. In the process of forming the outer diameter section, as the roll feed decreases, the roundness deviation will decrease accordingly. The difference of roundness deviation on different sections is particularly obvious at low rotational speeds. When the rotational speed is high enough, the values and deviations at different sections are low. Therefore, it is recommended to take the rotational speed of roll above 20 rpm in rolling the internal and external diameter sections.

#### 4.2.2. Chuck Axial Speed

**Figure 10:**
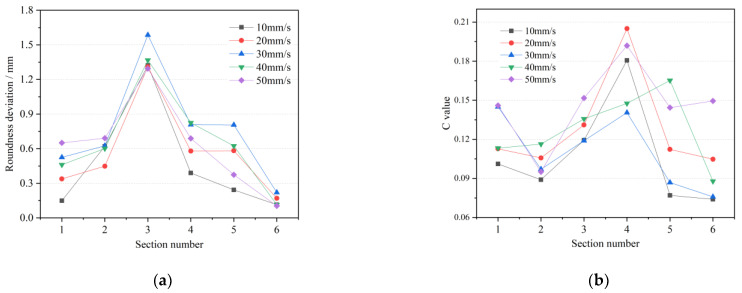
Forming quality under different chuck axial speeds (**a**) roundness deviation; (**b**) wall thickness uniformity.

It can be seen from Figure 10 that the variation trend of roundness deviation and wall thickness uniformity of the variable-diameter section under different chuck speeds are similar. The third section of the internal variable-diameter section of the former reaches the highest level, which conforms to the rule that the shape accuracy of the rolled piece decreases gradually with the decrease of the nominal diameter. The latter reaches the highest value in the fourth section except 40 mm/s. With the increase in axial speed, the outer roundness deviation of the first and second section increases gradually, but the increase is small. Even under the condition of large axial speed, the outer roundness deviation can still be controlled at about 0.7 mm, indicating that the section of the variable-diameter section in the rolled piece is close to the normal circle and the forming accuracy is high. The wall thickness uniformity of the inner diameter section is reduced, but the amplitude is very small. Even at the axial speed of 50 mm/s, the *C* value still can be maintained at 0.15. It shows that the chuck speed has little effect on the forming quality of the variable-diameter section. In the actual selection of process parameters, the axial speed of the rolling variable-diameter section can be appropriately increased, and the rolling process can be accelerated to reduce the heat loss and oxidation of the rolled piece under the premise of ensuring the *C* value of the section is maintained at a low level.

#### 4.2.3. Roll Feed Speed

**Figure 11:**
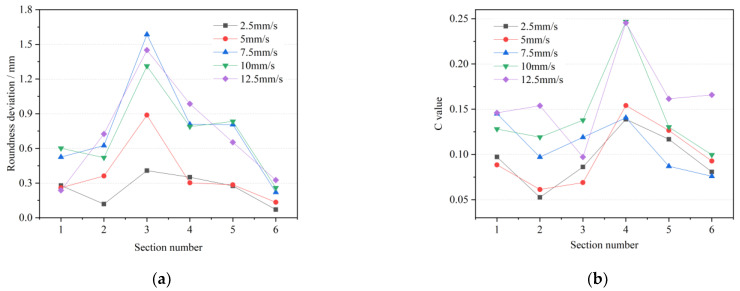
Forming quality under different roll feed speeds (**a**) roundness deviation; (**b**) wall thickness uniformity.

From Figure 11, it can be seen that the roundness deviation and *C* value of the variable-diameter section in the rolled piece increase with the increase in the roll feed speed. When the feed speed is greater than 7.5 mm/s, the deviation value on the third section is more than 1.2 mm, indicating that the shape of the outer profile section is not close to the positive circle, and there may be defects such as spiral lines or incomplete rolling. In addition, the fourth section of the *C* value at the speeds of 10 mm/s and 12.5 mm/s has mutations. Therefore, when the actual process parameters are selected, because the length of the variable-diameter section accounts for a small proportion of the whole axle, under a certain process combination, reducing the processing speed in order to improve the accuracy does not significantly affect the processing time of the whole axle. It can be considered to improve the roll radial feed speed without the occurrence of spiral marks, so as to maintain the uniformity of the cross-section wall thickness and reduce the rolling time as much as possible.

### 4.3. One-Way ANOVA Analysis

ANOVA is a statistical method for comparing the means of multiple samples and is designed to investigate whether factors have a significant effect on the response. If only one controllable factor is studied for its effect on the test results at different levels, this is a one-way ANOVA. One-way ANOVA was applied to the data in Figure 9, Figure 10 and Figure 11, where *SS_A_*, *SS_E_*, *MS_A_* and *MS_E_* are the sum of squared deviations between groups, the sum of squared deviations within groups, the variance between groups and the variance within groups, respectively, as in Equations (12)–(15) [30].

(12) $$SS_{A}=\sum_{i=1}^{m}{\left({\bar{x}}_{i}-\bar{\bar{x}}\right)}_{2}$$

(13) $$SS_{E}=\sum_{i=1}^{m}\sum_{j=1}^{n}\left(x_{ij}-{\bar{x}}_{i}\right)$$

(14) $$MS_{A}=\frac{SS_{A}}{m-1}$$

(15) $$MS_{E}=\frac{SS_{E}}{N-m}$$

where m=5, n=6, N=5×6=30. *x_ij_* is the value of any one result, $\bar{\bar{x}}$ is the total mean of the test results, ${\bar{x}}_{i}$ is the mean of the test results within any group, m−1 is the DOF (degree of freedom) of the $MS_{A}$, N is the total number of samples, i.e., m×n and N−m is the DOF of the $MS_{E}$.

The *F*-values were calculated and compared with the test levels to explore their significance on the response. If $F\geq F_{0.01}$, the factor is judged to be highly significant; if $F_{0.05}\leq F<F_{0.01}$, it is judged to be significant; if $F<F_{0.05}$, the factor is considered insignificant. The results of the one-way ANOVA are shown in Table 2, which shows that the roll rotational speed has a significant effect on the roundness deviation, and the roll feed speed is significant on the wall thickness uniformity, but neither of them reaches the highly significant level.

## 5. Process Parameters Optimization of Variable-Diameter Section

### 5.1. Design of Orthogonal Experiment Scheme

In order to explore the interaction effect of multiple interference factors on the forming quality of the specimen in the variable-diameter section of the three-roll skew rolling, the orthogonal experiment with five factors and four levels (Table 3) was designed to study the weight of the influence degree of the outer surface accuracy deviation and the roundness deviation of the specimen in the variable-diameter section of the hollow axle formed by the three-roll skew rolling of 30 CrMoA alloy steel at the multiple factor level. The parameter indexes of the surface accuracy and the roundness deviation were established for quantitative analysis, and the optimal process parameter combination under the best surface forming quality was obtained.

The surface precision deviation of the variable-diameter section reflects the deviation value between the surface point of the and the ideal surface point, which reflects the surface accuracy and is one of the evaluation criteria for evaluating the surface forming quality of the variable-diameter section. In the orthogonal experiment, specific measurement values are needed to reflect the overall forming accuracy of the whole rolled piece. The position deviation of the sampling point can be obtained by the difference between the coordinate value in the *x* or *y* direction and the coordinate value of the theoretical position. *d_i_* is the nominal diameter of the sampling point in the quadrant direction, and the average deviation $\bar{d}$ of all points in the variable-diameter section is calculated. *y_m_* is the ideal machining curve of the die end point, and Equations (14) and (15) are the ideal first-order functions used in the internal and external variable-diameter sections, respectively.

(16) $$d_{i}=2\sqrt{\left({x_{i}}^{2}+{y_{i}}^{2}\right)}$$

(17) $$\bar{d}=\frac{\sum_{i=n}^{n}\left|d_{i}-y_{i}\right|}{n}$$

(18) $$y_{m}=-0.266x_{m}+7.064$$

(19) $$y_{n}=0.267x_{n}+13.701$$

where the absolute transverse and ordinate values of sampling point *d_i_* are *d_x_* and *d_y_*. *x_m_* and *y_m_* are the transverse and longitudinal coordinates of the points on the ideal forming curve of the internal variable-diameter section and *x_n_* and *y_n_* of the external variable-diameter section.

The roundness deviation of the variable-diameter section is measured by the deviation of the radius or nominal diameter of each point on the section. The roundness deviation obtained by the minimum area method refers to the radius difference of the two concentric circles that contains the minimum radius difference of the measured circle contour as the roundness error. By calculating the nominal radius *R_i_*, the roundness deviation of each section on the variable-diameter section is averaged, which reflects the forming quality of the variable-diameter section of the rolled piece.

(20) $$R_{i}=\sqrt{{x_{i}}^{2}+{y_{i}}^{2}}$$

(21) $$\bar{f_{\phi }}=\frac{\sum f_{\phi }}{n}=\frac{\sum \left(R_{\max}-R_{\min\phi }\right)}{n}$$

where $\bar{f_{\phi }}$ is average roundness deviation, $f_{\phi }$ is roundness deviation and *x_i_* and *y_i_* are the transverse and longitudinal coordinates of the section points.

The L16 orthogonal table was selected, and the scheme configuration shown in Table 4 can be obtained from the factor level and orthogonal table. Through the finite element simulation, the influence of the above factors on the surface accuracy deviation and roundness deviation can be obtained to optimize the process parameters [31].

### 5.2. Range Analysis of Orthogonal Results

The orthogonal test can use the range analysis method to analyze the results. This method has strong practicability and simple calculation. It is the most commonly used method for orthogonal experimental analysis. The purpose is to find out the primary and secondary order of the influence of various process parameters on the forming accuracy of the outer surface of the TRSR hollow axle variable-diameter sample and the deviation of the outer roundness of the variable-diameter section, and obtain the optimal process combination for forming. The calculation is as follows:

(22) $$T_{i}=\sum_{i=1}^{n}x_{i}$$

(23) $$R=\max(T_{1},T_{2},T_{3},T_{4})-\min(T_{1},T_{2},T_{3},T_{4})$$

In the formula, *T_i_* represents the test index sum corresponding to the level number *i* (*i* = 1, 2, 3, 4) in any column, *t_i_* is the average value of the test index, *r* is the number of occurrences of each level in any column and *R* is the difference between the maximum and minimum values of the test index for each level of the factor in any column.

#### 5.2.1. Range Analysis of Surface Accuracy Deviation

The range analysis table of surface forming precision in variable-diameter section can be obtained by calculating and analyzing a series of numerical values of surface precision variation in orthogonal experiment results, as shown in Table 5.

In the table above, *R* is the range, which reflects the variation range of test indexes when each factor level changes. The greater the R value, the greater the impact of this factor on the test indicators. According to the *R* value of each factor, the primary and secondary order of each factor is B > C > D > A > E, that is, the roll rotational speed has the greatest influence on the surface forming accuracy, followed by the axial speed of the chuck, and then the radial velocity of the roll and the rolling temperature, whereas the roll feed angle has the smallest influence on the surface forming accuracy of the variable-diameter section.

The optimal level of factors can be judged by the minimum value of *T_i_* in the table, so A_4_B_4_C_2_D_4_E_3_ is the best level combination for surface forming accuracy. Above all, the surface forming accuracy of the hollow axle variable-diameter section sample is the highest when the rolling temperature is 1200 °C, the rotational speed of roller is 25 rpm, the axial traction speed of chuck is 20 mm/s, the radial feed speed of roller is 10 mm/s, and the feed angle of roller is 7°.

#### 5.2.2. Range Analysis of Outer Roundness Deviation

The range analysis of outer roundness deviation shown in Table 6 can be obtained by calculating and analyzing the outer roundness deviation of rolling section. The *R* value analysis is the same as Table 5. The influence order of various factors on the outer roundness is B > D > A > E > C, and the optimal level combination is A_3_B_4_C_2_D_4_E_2_. It can be seen that the roller speed has the greatest influence on the surface forming quality, and reaches the optimal level at 25 rpm.

### 5.3. ANOVA and Correlation Analysis

In order to accurately estimate the significance of the test results of each factor, the orthogonal test results were verified by ANOVA analysis. No empty column was set in the orthogonal test table of L_16_ (4^5^). A factor with the smallest sum of squares was used as the error, and the mean square deviation of *MS_j_* < *2MSE* was added to the sum of squares of the error. The DOF of factor *j* was also added to the error, which was expressed by * in the Table 7. The calculation method of deviation sum of squares, mean square deviation and *F* value was the following [30].

Total deviation square sum, deviation square sum of each column and error square sum:

(24) $$SST=\sum_{i=1}^{n}{\left(x_{i}-\bar{x}\right)}^{2}=\sum_{i=1}^{n}{x_{i}}^{2}-\frac{T^{2}}{n}$$

(25) $$SSj=r\sum_{i=1}^{n}{\left(t_{i}-\bar{x}\right)}^{2}=\frac{1}{r}\sum_{i=1}^{m}{T_{i}}^{2}-\frac{T^{2}}{n}(j=1,2,\ldots k)$$

(26) $$SSE=SST-\sum_{j=1}^{k}SSj$$

Total degree of freedom, degree of freedom of each factor and error degree of freedom:

(27) $$f_{T}=n-1,\;f_{j}=m-1,\;f_{e}=f_{T}-\sum_{j=1}^{k}f_{j}$$

Value of *F*:

(28) $$F_{j}=MS_{j}/MSE$$

In the analysis of variance, it is necessary to compare the corresponding *F* value with the critical value of each significant level to see whether the influence of each factor on the test is significant or not. If factor *F* exceeds the threshold of a certain level of aboriginality, then this factor is aboriginal at this level of aboriginality; on the contrary, this factor has no aboriginal characteristics at this level. The ANOVA analysis of Table 4 is shown in Table 7, and *F*_0.05_ (3, 15) = 3.290 and *F*_0.01_ (3, 15) = 5.417. It can be seen that only the main effect of roll speed on the outer roundness deviation is obvious (# in the table). The primary and secondary factors can be arranged according to the *F* value. In Table 6, whether the surface accuracy deviation or outer roundness deviation is used as the evaluation index, the *F* value of B factor is the largest, which shows that the roll speed has the most obvious effect on the forming quality of variable-diameter section of hollow axle in three-roll skew rolling. In the range analysis above, *R_B_* is also the maximum, which is consistent with the results of Section 5.2.

To explore the correlation between two responses, the Pearson correlation coefficient was used. It is defined as the quotient of the product of the covariance and standard deviation between two variables, and is often expressed as *r*.

(29) $$r=\frac{Cov(X,Y)}{\sigma_{x}\cdot \sigma_{y}}=\frac{\sum_{i=1}^{n}\left(x_{i}-\bar{x})(y_{i}-\bar{y}\right)}{\sqrt{\sum_{i=1}^{n}{\left(x_{i}-\bar{x}\right)}^{2}}\cdot \sqrt{\sum_{i=1}^{n}{\left(y_{i}-\bar{y}\right)}^{2}}}$$

The surface accuracy deviation is denoted by *X*, and the external roundness deviation is denoted by *Y* here, where $\bar{x}$, $\bar{y}$ denotes the sample mean. The calculated correlation coefficient was 0.9657, which shows that the correlation between the surface accuracy deviation and outer roundness deviation are positively and strongly correlated.

## 6. Conclusions

(1) The reliability of the finite element model was verified by comparing the FE and experimental results. The generation mechanism of the surface spiral mark defect and the spiral mark depth formula (maximum size deviation) of the variable-diameter section in the three-roll skew rolling process were explored by taking the deformed variable-diameter section sample as the research object. The maximum size deviation of internal diameter section *f_i_* and external diameter section *f_o_* are:
  $$f_{i}=\frac{z_{i}\tan\theta }{\tan\theta +1}=\frac{10dV\sin\theta }{Rn_{r}\cos^{2}\theta (tan\theta +1)}$$
  $$f_{o}=\frac{z_{o}\cdot \tan\theta \cdot \tan(\beta -\theta )}{\tan\theta +\tan(\beta -\theta )}=\frac{10dV\cdot \tan\theta \cdot \tan(\beta -\theta )}{Rn_{r}\left[\tan\theta +\tan(\beta -\theta )\right]}$$
(2) An index function *C* to measure the uniformity of cross-sectional wall thickness was established, and the influence laws of each process parameter on the roundness deviation and wall thickness uniformity were obtained by a single-factor simulation experiment and one-way ANOVA analysis. When actually selecting process parameters, it is recommended to select a roll rotational speed of more than 20 rpm. The axial speed can be appropriately increased to maintain the *C* value at a low level, which can accelerate the rolling process and reduce the heat loss and oxidation. Consider increasing the radial feed speed in the range of 2.5–7.5 mm/s without spiral trace defects, which can maintain the thickness uniformity and minimize the rolling time. The one-way ANOVA results show that roll rotational speed has a significant effect on the roundness deviation, and the roll feed speed is significant on the wall thickness uniformity.
(3) The interaction effect of multi-interference factors on the forming quality of the TRSR variable-diameter section was investigated. Orthogonal tests were designed and the order and optimal combination of process parameters on the forming quality were obtained by range analysis and ANOVA analysis. The results consistently show that the roll speed has the most significant effect on the surface forming quality. The surface accuracy deviation and roundness deviation are positively and strongly correlated.

## Figures and Tables

**Table 1:** Parameters setting of FEM single-factor experiment.

Factor	Roll Rotational Speed *n*/(rpm)	Chuck Axial Speed *v_A_*/(mm/s)	Roll Feed Speed *v_R_*/(mm/s)
1	10	10	2.5
2	20	20	5
3	30	30	7.5
4	40	40	10
5	50	50	12.5

**Table 2:** One-way ANOVA analysis.

Response	Roundness Deviation			Wall Thickness Uniformity		
Factor	*n*	*v_A_*	*v_R_*	*n*	*v_A_*	*v_R_*
$SS_{A}$	10.941	0.2777	1.3552	4.2516	0.0061	0.0205
$SS_{E}$	19.9238	4.5204	3.1144	12.2588	0.028	0.04
$MS_{A}$	2.7353	0.0694	0.3388	1.0629	0.0015	0.0051
$MS_{E}$	0.797	0.1808	0.1246	0.4904	0.0011	0.0016
F	3.432 *	0.384	2.719	2.167	1.364	3.188 *

*F*_0.05_(4, 25) =2.759, *F*_0.01_(4, 25) =4.177. *: $F_{0.05}\leq F<F_{0.01}$.

**Table 3:** Horizontal table of orthogonal test factors.

Level	Factor				
	Rolling Temperature *T*/(°C)	Roll Rotational Speed *n*/(rpm)	Chuck Axial Speed *v_A_*/(mm/s)	Roll Feed Speed *v_R_*/(mm/s)	Feed Angle *α*/(°)
1	1050	10	10	2.5	3
2	1100	20	20	5	5
3	1150	30	30	7.5	7
4	1200	40	40	10	9

**Table 4:** Test arrangements and results.

No	A	B	C	D	E	Surface Accuracy Deviation/mm	Outer Roundness Deviation/mm
1	1	1	1	1	1	4.121	3.727
2	1	2	2	2	2	0.524	0.731
3	1	3	3	3	3	0.432	0.515
4	1	4	4	4	4	0.227	0.146
5	2	1	2	3	4	0.861	1.202
6	2	2	1	4	3	0.522	0.500
7	2	3	4	1	2	0.624	0.337
8	2	4	3	2	1	0.165	0.195
9	3	1	3	4	2	1.140	0.624
10	3	2	4	3	1	0.846	0.949
11	3	3	1	2	4	0.474	0.335
12	3	4	2	1	3	0.105	0.144
13	4	1	4	2	3	0.917	1.272
14	4	2	3	1	4	0.651	0.972
15	4	3	2	4	1	0.082	0.075
16	4	4	1	3	2	0.261	0.304

**Table 5:** Range analysis of surface accuracy deviation.

Parameter	Surface Accuracy Deviation				
	A	B	C	D	E
*T*_1_	5.304	7.039	5.378	5.501	5.214
*T*_2_	2.172	2.543	1.572	2.08	2.549
*T*_3_	2.565	1.612	2.388	2.4	1.976
*T*_4_	1.911	0.758	2.614	1.971	2.213
*R*	3.393	6.281	3.806	3.53	3.238

**Table 6:** Range analysis of outer roundness deviation.

Parameter	Outer Roundness Deviation				
	A	B	C	D	E
*T*_1_	5.119	6.825	4.866	5.18	4.946
*T*_2_	2.234	3.152	2.152	2.533	1.996
*T*_3_	2.052	1.262	2.306	2.97	2.431
*T*_4_	2.623	0.789	2.704	1.345	2.655
*R*	3.067	6.036	2.714	3.835	2.95

**Table 7:** ANOVA analysis.

Factor	DOF	Surface Accuracy Deviation			Outer Roundness Deviation		
		*SS_j_*	*MS_j_*	*F_j_*	*SS_j_*	*MS_j_*	*F_j_*
A *	3	1.842	0.614	0.955	1.529	0.510	1.027
B	3	5.869	1.956	3.041	5.641	1.880	3.786 #
C *	3	2.054	0.685	1.065	1.193	0.398	0.801
D *	3	2.130	0.710	1.104	1.928	0.643	1.294
E *	3	1.693	0.564	0.877	1.309	0.437	0.879
Error	12	7.72	0.643		5.959	0.497	
Total	15	13.588			11.600		

*F*_0.05_(3, 15) = 3.290, *F*_0.01_(3, 15) = 5.417. *: *MS_j_* < *2MSE*. #: $F_{0.05}\leq F_{j}<F_{0.01}$.

## Data Availability

The data presented in this study are available on request from the corresponding author.
